# Astragaloside IV Targets Macrophages to Alleviate Renal Ischemia-Reperfusion Injury via the Crosstalk between Hif-1α and NF-κB (p65)/Smad7 Pathways

**DOI:** 10.3390/jpm13010059

**Published:** 2022-12-27

**Authors:** Lumin Tang, Minyan Zhu, Xiajing Che, Xiaoqian Yang, Yao Xu, Qing Ma, Ming Zhang, Zhaohui Ni, Xinghua Shao, Shan Mou

**Affiliations:** 1 Department of Nephrology, Ren Ji Hospital, School of Medicine, Shanghai Jiao Tong University, Shanghai 200127, China; 2 Department of Urology, Ren Ji Hospital, School of Medicine, Shanghai Jiao Tong University, Shanghai 200127, China

**Keywords:** Astragaloside IV, macrophage polarization, renal ischemia-reperfusion, renal fibrosis, NF-κB (p65)/Hif-1α, Hif-1α/Smad7

## Abstract

(1) Background: Astragaloside IV (AS-IV) is derived from Astragalus membranous (AM), which is used to treat kidney disease. Macrophages significantly affect the whole process of renal ischemia-reperfusion (I/R). The regulation of macrophage polarization in kidneys by AS-IV was the focus. (2) Methods: Renal tubular injury and fibrosis in mice were detected by Hematoxylin and Eosin staining and Masson Trichrome Staining, separately. An ELISA and quantitative real-time polymerase chain reaction were used to explore the cytokine and mRNA expression. Western blot was used to determine protein expression and siRNA technology was used to reveal the crosstalk of signal pathways in RAW 264.7 under hypoxia. (3) Results: In the early stages of I/R injury, AS-IV reduced renal damage and macrophage infiltration. M1-associated markers were decreased, while M2 biomarkers were increased. The NF-κB (p65)/Hif-1α pathway was suppressed by AS-IV in M1. Moreover, p65 dominated the expression of Hif-1α. In the late stages of I/R injury, renal fibrosis was alleviated, and M2 infiltration also decreased after AS-IV treatment. Hif-1α expression was reduced by AS-IV, while Smad7 expression was enhanced. Hif-1α interferes with the expression of Smad7 in M2. (4) Conclusions: AS-IV promoted the differentiation of M1 to M2, relieving the proinflammatory response to alleviate the kidney injury during the early stages. AS-IV attenuated M2 macrophage infiltration to prevent kidney fibrosis during the later stages.

## 1. Introduction

Acute kidney injury (AKI) is considered a major clinical emergency. The prevalence of AKI in the intensive care unit (ICU) has reportedly reached 50%. AKI may lead to poor short- or long-term outcomes, which can increase the risk of chronic kidney disease (CKD) and end-stage renal disease (ESRD). Ischemia-reperfusion injury (IRI) represents the critical factor in inducing AKI, which causes renal impairment, and renal fibrosis in the long-term. Nevertheless, effective therapeutic measures for kidney injury are still limited. For many decades, renal replacement therapy (RRT) has been the only feasible treatment [1,2,3,4].

Astragaloside IV (AS-IV) is the primary active component of Astragalus membranaceus (AM). It is usually prescribed alone or with other herbs to treat kidney diseases in clinics of traditional Chinese medicine. Previous studies have indicated that AM mainly works through its anti-inflammatory, antifibrosis, and antioxidant activity, clinically reducing proteinuria and restoring renal function [5]. However, the precise mechanism by which AS-IV protects against kidney injury has not been entirely elucidated yet.

Macrophages have important effects on the entire IRI process, such as injury, inflammation, repair, and fibrosis [6]. The M1 macrophage is called the “classical activated macrophage”. It appears in the injury areas after 24–48 h of ischemia-reperfusion (I/R) and infiltrates into the tubular tissue, promoting kidney damage through immune responses such as releasing inflammatory cytokines and interleukins during the early stages. The M2 macrophage is known as an “alternatively activated macrophage”, which inhibits inflammation and promotes tissue repair, but induces renal fibrosis mainly in the later stages of kidney injury [7]. Regulating the M1/M2 subtype transformation may alter the prognosis of renal injury [8,9].

Therefore, we hypothesized that AS-IV intervenes in kidney injury outcomes by acting on immune cells. AS-IV could modulate differentiation and infiltration of renal macrophages to mitigate inflammatory response and fibrosis during the process of renal IRI.

## 2. Materials and Methods

### 2.1. Study Design

**Figure 1:**
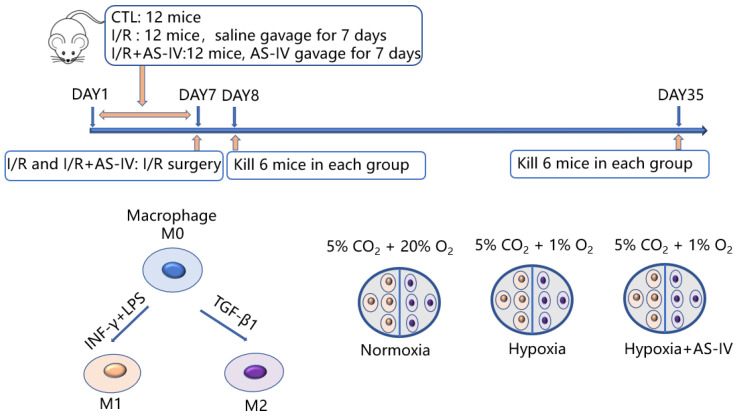
Schematic flowchart of the experimental design.

In this study, there were in vitro and in vivo experiments as shown in Figure 1. In vivo, 36 mice were later classified into three groups: I/R, I/R+AS-IV, and control. Both I/R and I/R+AS-IV groups underwent I/R surgery with bilateral renal pedicles being clamped for 30 min. Before the I/R operation, the mice in the I/R+AS-IV group were gavaged for 7 consecutive days with AS-IV (20 mg/kg/day, 84687-43-4, Sigma-Aldrich, MO, USA), and the mice in I/R group were gavaged with saline. Mice were euthanized after 24 h or 28 days of I/R operation. In vitro, we classified RAW 264.7 cells into three groups: the normoxia group cultured at 5% CO_2_ and 20% O_2_; the hypoxia group cultured at 1% O_2_ and 5% CO_2_; and the AS-IV exposed hypoxia group cultured at 1% O_2_ and 5% CO_2_ for 24 h. 

### 2.2. Animals

Male C57BL/6 mice, aged 6–8 weeks old, were purchased from the Shanghai Laboratory Animal Center (Chinese Academy of Sciences). All animal experiments conducted were carried out in accordance with the internationally accepted guidelines for the care and use of laboratory animals. Mice were maintained at constant temperature (24 ± 1 °C) with 12 h light/12 h dark cycle and 40% relative humidity. All animals were allowed to eat standard chow and drink water freely.

### 2.3. Cell Culture and Treatment

RAW 264.7 cells were obtained from the American Type Culture Collection and cultivated in a high-glucose (21063029, HG/DMEM, Gibco, MA, USA) medium containing 10% fetal bovine serum (FBS, 16140071, Gibco, MA, USA) at 37 °C in 5% CO2 conditions. RAW 264.7 cells were stimulated with recombinant IFN-γ (20 ng/mL, NP032363, R&D Systems, MI, USA) and LPS (20 ng/mL,297-473-0, Sigma-Aldrich, MO, USA) for 24 h until they underwent M1 polarization; another portion of the RAW 264.7 cells was incubated for 24 h following the addition of TGF-β1 (20 ng/mL, P04202 R&D Systems, Minneapolis, USA) to induce M2 differentiation. 

### 2.4. Histological Examination

The hematoxylin and Eosin Staining Kit (60524ES60, Yeasen, Shanghai, China) was used according to the manufacturer’s instructions. For the presence of morphologic changes, tubular injury was scored as previously reported [10]. We evaluated the pathology score with two senior pathologists on a blind basis. Each kidney section was analyzed in at least ten fields.

To assess severity of kidney fibrosis, Masson’s trichrome was performed with Masson Stain Kit (G1006, Servicebio, Wuhan, China). It was scored as the percentage of tubulointerstitial fibrosis: no fibrosis (0), less than 25% (1), 25–50% (2), 50–75% (3), and more than 75% (4).

An immunohistochemistry assay was conducted in accordance with standard protocols. F4/80 (1:500, ab300421, Abcam, MA, USA) or α-SMA (1:300, sc-53142, Santa Cruz, CA, USA) were applied to paraffin-embedded sections. Quantification was calculated as a percentage of areas stained positively to total areas.

In the immunofluorescence assay, macrophages were identified with primary antibodies α-SMA (1:500, sc-53142, Santa Cruz, CA, USA) and CD206 (1:200, ab300621, Abcam, Cambridge, UK). 

### 2.5. Cytokine Assays 

As instructed by the manufacturer, ELISA kits were used to detect IL-10 (EK210, Multi Sciences, Hangzhou, China), TNF-α (EK282, Multi Sciences, Hangzhou, China), and IL-6(DY406-05, R&D Systems, Minneapolis, USA).

### 2.6. Western Blot Analysis 

Protein was extracted from kidney tissues and RAW 264.7 cells. Protein concentrations were determined using the BCA Protein Assay Kit (71285-M, Millipore, Darmstadt, Germany). In 6%, 7.5%, and 12% SDS-PAGE gels, equal amounts of protein (30 μg/lane for RAW 264.7 cells and 60 μg/lane for kidney tissues) were separated. Subsequently, they were transferred onto nitrocellulose membranes. They were then blocked for 2 h in 5% skim milk, and 4 °C incubation was carried out overnight on the membranes with primary antibodies for Kim-1- (1:1000, ABF199, Sigma Aldrich, Darmstadt, Germany), β-actin (1:5000, 3700, Cell Signaling Technology, MA, USA), Hif-1α (1:1000, 36169, Cell Signaling Technology, MA, USA), p65 (1:2000, 6956, Cell Signaling Technology, MA, USA), arginase-1 (1:500, sc-18354, Santa Cruz, CA, USA), fibronectin (1:1000, ab45688, Abcam, Cambridge, UK), collagen 1 (1:1000, ab260043,Abcam, MA, USA), smad7 (1:500, sc-365846, Santa Cruz, CA, USA), and α-tubulin (1:2000, sc-8035, Santa Cruz, CA, USA). Antirabbit or antimouse second antibodies conjugated to horseradish peroxidase were added to the membranes for one hour. Protein bands were visualized by chemiluminescence detection, while the signal intensities were analyzed using Image J software (NIH, Bethesda, MD, USA). 

### 2.7. RNA Preparation and qPCR

Trizol reagent (15596026, Invitrogen, Carlsbad, CA, USA) was utilized for the extraction of the total RNA. Next, 1000 ng total RNA was reverse transcribed into cDNA using the RevertAid First Strand cDNA Synthesisit (K1622, Invitrogen, Carlsbad, USA. Quantity Nova SYBR Green PCR Kit (208054, QIAGEN, Hilden, Germany) was adopted for qPCR, with β-actin used as an endogenous reference. The PCR amplification was performed using the primers mentioned in Table 1(Sangon Biotech, Shanghai, China). The ∆∆Ct approach was used to determine the gene expression levels. 

### 2.8. Knockdown of NF-κB (p65)/Hif-1α/Smad7 by Small Interfering RNA (siRNA) 

For silencing the Hif-1α gene, we prepared the negative control (si-nc) and Hif-1α-specific siRNA (si-Hif-1α) using a chemical method (Genomeditech, Shanghai, China). Next, the RAW 264.7 cells were inoculated overnight in 6-well plates, and the cells were transfected with the nontargeting control or sequence-specific siRNA using Lipofectamine 3000. The transfection was performed as per specific instructions. After 6 h of transfection, the medium (Opti-MEM) was changed, and after 24 h, cells were analyzed. The transfection of NF-κB (p65)-specific siRNA and Smad7-specific siRNA were also performed as per the above description.

### 2.9. Statistical Analysis

GraphPad Prism 9 (version 9.0.0, GraphPad Software Inc. San Diego, CA, USA) was used for data analysis. Data were expressed as mean and standard deviation (mean ± SD). To analyze the difference between the two groups, Student’s *t*-tests were used. *p* < 0.05 or *p* < 0.01 was determined as a statistical significance for all data.

## 3. Results 

### 3.1. AS-IV Reduced the Acute Renal Injury 24 h after Ischemia-Reperfusion In Vivo

**Figure 2:**
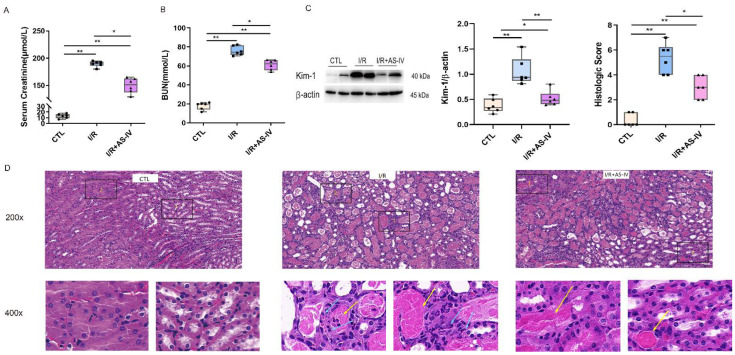
Astragaloside IV reduces acute renal injury induced by ischemia-reperfusion. (**A**,**B**) The level of serum creatinine and urea nitrogen 24 h after ischemia-reperfusion. (**C**) Western blotting shows the expression of kidney injury molecule-1 (Kim-1) in kidney tissue 24 h after ischemia-reperfusion. (**D**) Representative H&E staining of renal tissue sections 24 h after ischemia-reperfusion (magnification ×200, ×400); blue arrows indicate tubular epithelial cell necrosis with nuclear dissolution or fragmentation and calcification. Yellow arrows indicate eosinophils infiltration. n = 6 in each group, * *p* < 0.05, ** *p* < 0.01.

The focus of this study is the role of AS-IV in protection against AKI caused by ischemia-reperfusion. Based on previous research, we selected post 24 h I/R as the critical time for observation. Compared with the CTL group, the I/R and I/R+AS-IV groups had elevated serum urea nitrogen and creatinine. Compared with the IR+AS-IV group, these contents had a marked increase in the I/R group (Figure 2A,B). Based on the above findings, we found that AS-IV decreased serum creatinine and urea nitrogen levels to protect against acute renal injury. 

Kidney injury molecule-1 (Kim-1) is a biomarker of acute kidney injury, with its expression levels reflecting the degree of renal tubular injury. Western blot results showed that the Kim-1 protein was rarely expressed in normal mice but highly expressed in kidney tissues of mice after I/R. After the intervention of AS-IV, the expression of Kim-1 significantly decreased (Figure 2C).

In renal pathology, the HE staining showed that the CTL group had a clear division of the renal medullary cortex. It also showed a clear structure of glomerular capillaries along with a normal structure of renal tubular epithelial cells in the CTL group. The I/R group had obvious tubular necrosis, especially epithelial cell necrosis and shedding along with nuclear dissolution or fragmentation and calcification after partial necrosis. This was accompanied by large amounts of inflammatory cell infiltration along with the filling of the lumen with eosinophils in the renal tubular cortex and medulla. The IR+AS-IV group also had tubular necrosis, epithelial cell necrosis, shedding, and nuclear dissolution or fragmentation but to a less severe extent. However, inflammatory cell infiltration was rare, with no obvious necrotic calcification or eosinophils observed in the lumen of the renal tubulointerstitium (Figure 2D). Pathologically, we found that AS-IV could reduce acute kidney injury caused by the I/R.

### 3.2. AS-IV Inhibited the Infiltration of Macrophages and Promoted the Phenotypic Switching from M1 to M2 24 h after Ischemia-Reperfusion In Vivo

**Figure 3:**
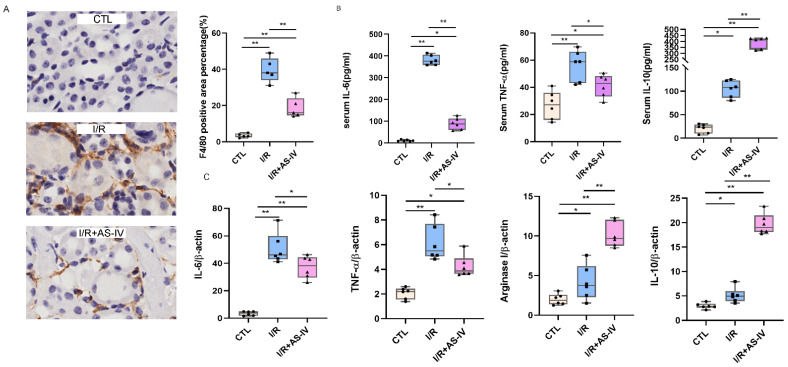
AS-IV inhibits macrophage infiltration and promotes M1 to M2 phenotypic switching 24 h after I/R. (**A**) Immunohistochemistry staining of F4/80 and quantification score of CTL, I/R, I/R+AS-IV groups (magnification ×400). The brown areas represent positive expression of F4/80. (**B**) Serum IL-10, TNF-α, and IL-6 levels 24 h after ischemia-reperfusion determined by ELISA. (**C**) Relative mRNA levels of IL-6, TNF-α, Arginase I, and IL-10 in kidney tissues. *n* = 6 in each group, * *p* < 0.05, ** *p* < 0.01.

To study the role of macrophages in IRI, immunohistochemical staining was used to evaluate the infiltration of macrophages in the kidneys of mice after 24 h of I/R. The macrophages are labelled with F4/80 and the diagram shows a brownish-yellow color. As shown in Figure 3A, almost no interstitial tubules appear brownish yellow in the control group. However, more brownish-yellow areas were found in the I/R group than in the I/R+AS-IV group. This indicates that macrophage infiltration was reduced in renal tissues by AS-IV.

Macrophages belong to the innate immune cells, which participate in the inflammatory response after the occurrence of acute kidney injury. M1 macrophages secrete IL-6 and TNF-α, while M2 macrophages produce IL-10. According to the ELISA results, the IL-6, IL-10, and TNF-α contents in the I/R and I/R+AS-IV groups were higher than those of the CTL group. However, compared with the I/R group, AS-IV could significantly reduce the secretions of TNF-α and IL-6, while up-regulating the IL-10 expression in the I/R+AS-IV group (Figure 3B). Moreover, their mRNA expression exhibited the same trend within the renal tissues (Figure 3C). Our data revealed that AS-IV inhibited macrophage infiltration and promoted the phenotypic switch from M1 to M2.

### 3.3. AS-IV Promoted Macrophage Polarization toward M2 through the Suppression of NF-κB p65/Hif-1α Pathway In Vitro

**Figure 4:**
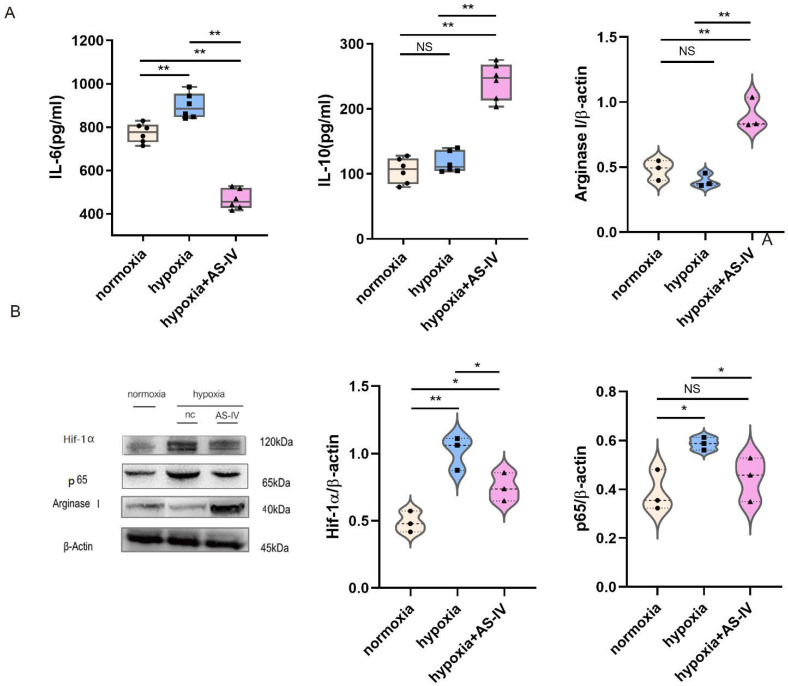
AS-IV promotes macrophage polarization toward M2 phenotype through suppressing NF-κB p65/Hif-1α pathway in vitro. (**A**) Levels of IL-6 and IL-10 in M1 cellular supernatants was analyzed by ELISA. (**B**) Western blot results of Arginase I, p65, and Hif-1α in RAW 264.7 under normoxia, hypoxia, or hypoxia with AS-IV; *n* = 3 in each group, NS, no significance, * *p* < 0.05, ** *p* < 0.01.

During the early stages of IRI, M1 forms the predominant macrophage that infiltrates the kidney tissues. To investigate the regulatory mechanism of AS-IV promoting macrophage phenotypic switching further, M1 was established using both IFN- and LPS. In a hypoxic environment, M1 secreted significantly more IL-6 than in a normoxic environment. Compared with the normoxic environment, IL-6 secreted by M1 in a hypoxic environment significantly increased. After intervention with AS-IV, IL-6 decreased significantly, while IL-10 secreted by M2 increased. AS-IV induced M1 to differentiate into M2 under hypoxia. (Figure 4A) Activation of the NF-κB (p65) signaling pathway in the macrophages can promote the activation of M1 macrophages to secrete proinflammatory factors, aggravating inflammatory damage. In addition, the expression of Hif-1α was elevated to cope with the hypoxic environment. We also found that, compared with normoxic conditions, the expression of Hif-1α significantly increased under hypoxic conditions. The expressions of both Hif-1α and p65 showed significant decreases after the intervention of AS-IV. Interestingly, the level of Arginase I, the marker of M2 activation, was found to be lower in M1 under both normoxic and hypoxic conditions, yet significantly higher under hypoxia with AS-IV (Figure 4B).

### 3.4. Hif-1α Was Mediated by p65 in M1 Macrophage under Hypoxic Conditions In Vitro 

**Figure 5:**
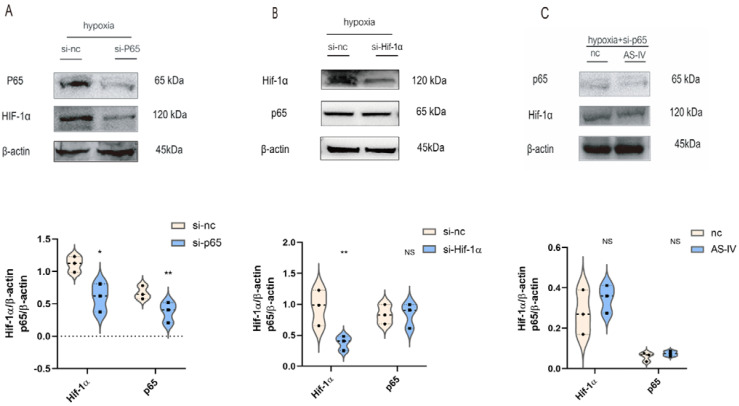
AS-IV regulated Hif-1α is mediated by p65 under hypoxia. (**A**) Western blotting analysis of p65 and Hif-1α after transfection of p65 siRNA or si-nc. (**B**) Western blotting analysis of p65 and Hif-1α after transfection of Hif-1α siRNA or si-nc. (**C**) Western blotting analysis of p65 and Hif-1 after transfection of p65 siRNA with or without AS-IV; *n* = 3 in each group, NS, no significance, * *p* < 0.05, ** *p*< 0.01.

To illustrate the interaction between p65 and Hif-1α, M1 cells were transfected with p65 or Hif-1α-siRNA sequence, respectively. In hypoxic conditions, the expression of p65 was significantly reduced after the siRNA treatment, which was accompanied by the low expression of Hif-1α (Figure 5A). However, the expression of p65 did not change significantly after the knockdown of Hif-1α (Figure 5B). NF-κB p65 regulated the expression of Hif-1α in M1 under hypoxic conditions. Upon silencing p65 under a hypoxic environment, no significant difference was observed in the levels of Hif-1α in the presence and absence of AS-IV (Figure 5C). This demonstrated that the inhibitory effects of AS-IV on Hif-1α were mediated by p65 under hypoxic conditions. 

### 3.5. AS-IV Attenuated Kidney Fibrosis 28 Days after the Renal Ischemia-Reperfusion Injury In Vivo

**Figure 6:**
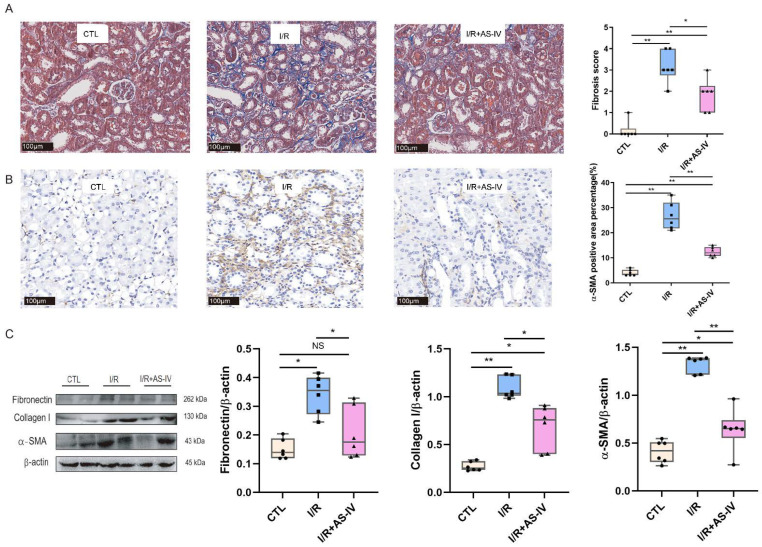
AS-IV attenuates kidney fibrosis 28 days after I/R. (**A**) Representative Masson staining (blue area) of renal tissue sections (magnification ×200). (**B**) Immunohistochemistry staining of α-SMA. The brown areas represent positive expression of α-SMA (magnification ×200). (**C**) Western blot results of fibrotic proteins (Fibronectin, Collagen I, and α-SMA) in renal tissues 28 days after I/R. *n* = 6 in each group, NS, no significance, * *p* < 0.05, ** *p* < 0.01.

We further observed whether the status of kidney fibrosis, as a subsequent result of tubular injury and inflammatory infiltration, could be changed by AS-IV in the long-term prognosis of renal IRI. We performed Masson staining and immunohistochemistry staining of α-SMA for this purpose. We found that AS-IV could reduce kidney fibrosis after 28 days of I/R operation (Figure 6A,B). The I/R+AS-IV group had a reduced expression of fibrotic proteins (Fibronectin, Collagen I, and α-SMA) in renal tissues compared with the I/R group (Figure 6C).

### 3.6. AS-IV Reduced the Infiltrated M2 and Fibrosis in the Renal Interstitium 28 Days after the Ischemia-Reperfusion Injury

**Figure 7:**
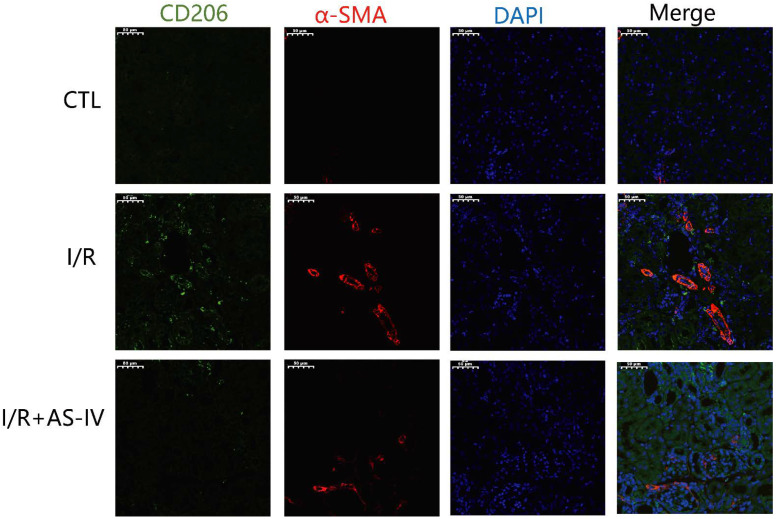
AS-IV reduces infiltrated M2 macrophages in the renal interstitium 28 days after I/R. Representative immunofluorescent co-staining images of CD206 and α-SMA in the renal interstitium (magnification ×200). Green corresponds to CD206, red corresponds to α-SMA, and blue corresponds to nuclear staining (DAPI).

M2 macrophages have been reported to be the main cell population of the macrophages present in the later stages of I/R injury. These macrophages induce tissue fibrosis by increasing the expression of TGF-β1. As shown in Figure 7, we also found CD206+ macrophages on the 28th day after I/R operation. Furthermore, we observed that the I/R group had more CD206+/α-SMA+ double-positive cells in the renal tubulointerstitium than the I/R+AS-IV group. In contrast, the CTL group had no positive cells. 

### 3.7. AS-IV Regulated M2 through the Hif-1α/Smad7 Signaling Pathway In Vitro

**Figure 8:**
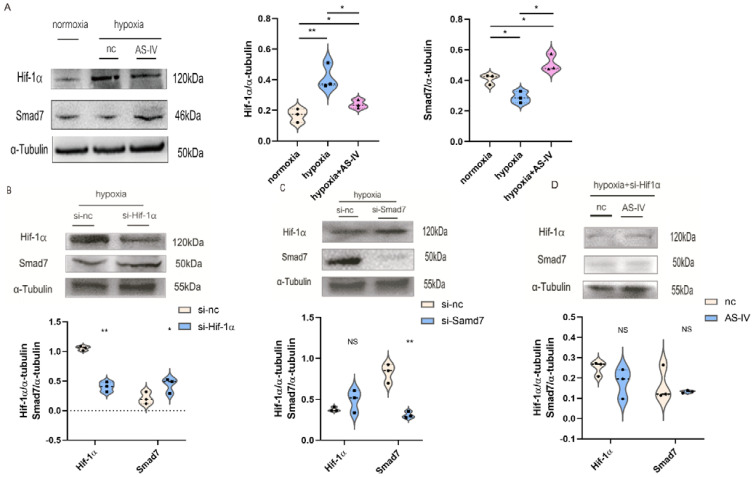
AS-IV regulates M2 macrophages through Hif-1α/Smad7 signaling pathway. (**A**) Western blot results of Hif-1α and Samd7 in M2 macrophages under normoxia, hypoxia, hypoxia with AS-IV. (**B**) Western blotting results of Smad7 after transfection of Hif-1α siRNA or si-nc in M2 macrophages under hypoxic conditions. (**C**) Western blotting results of Hif-1α after transfection of Smad7 siRNA or si-nc in M2 macrophages under hypoxic conditions. (**D**) Western blot results of Hif-1α and Samd7 in M2 macrophages transfected of Hif-1α siRNA under hypoxic conditions with or without AS-IV. *n* = 3 in each group. NS, no significance, * *p* < 0.05, ** *p* < 0.01.

To further investigate the regulatory mechanism of AS-IV in M2, we performed a Western blot to detect Hif-1α and Smad7 proteins. Activation of the TGF-β1/Smad7 signaling pathway in macrophages activated M2 macrophages, which contributed to the secretion of anti-inflammatory cytokines while promoting proliferation, regeneration, and sometimes fibrosis during chronic inflammatory responses. The expression of Hif-1α was elevated to cope with the hypoxic environment. At the later stages of IRI, the predominant macrophages infiltrating the kidney tissues were found to be M2. Therefore, TGF-β1 was used to induce the establishment of M2. Next, we found that a significant increase was observed in the expression of Hif-1α under hypoxic conditions compared with normoxic conditions. However, after the intervention of AS-IV, a significant decrease was observed in the levels of Hif-1α. There was also a decrease in Smad7 expression under hypoxic conditions; however, after AS-IV intervention, there was a significant increase in Smad7 levels (Figure 8A). The siRNA sequences for Hif-1α and Smad7 were separately transfected into the RAW 264.7 cells, which were then polarized to the M2 phenotype. In hypoxic conditions, the expression of Hif-1α was significantly reduced after the siRNA treatment, accompanied by high expression levels of Smad7 (Figure 8B). Upon the knockdown of Smad7, Hif-1α did not show significant changes (Figure 8C). This revealed that Hif-1α was a regulator of Smad7 in RAW 264.7 cells under hypoxic conditions. Interestingly, upon silencing the expression of Hif-1α under the hypoxic environment, no significant difference was observed in the Smad7 protein in the presence and absence of AS-IV (Figure 8D). This demonstrated that AS-IV’s promoting effects on Smad7 were mediated by Hif-1α under hypoxic conditions.

## 4. Discussion

The pathophysiological basis of ischemia-reperfusion- induced acute kidney injury is complex. The inflammatory cascade involves chemokines, cytokines, the expression of adhesion molecule, leukocyte infiltration and activation, the generation of oxidative stress and lipid peroxidation, mitochondrial dysfunction, and nitrite and nitric oxide production; all of these factors lead to cell death and renal damage [11]. Simultaneously, the immune cells (i.e., the T cells, macrophages, and dendritic cells) migrate to the site of injury, directly mediating the process of disease [12]. Previous studies have demonstrated that macrophages are highly heterogeneous and plastic in nature. They can differentiate into specific phenotypes to cope with the changes, which are controlled by the microenvironment [5]. M1 mainly causes inflammatory responses, which elicit subsequent damage to the kidneys during the primary stages. Conversely, the function of M2 is anti-inflammatory wound healing and follow-up fibrosis [13]. Early-stage proinflammatory M1 infiltration contributes to the worsening of renal injury. Other researchers found that when the macrophage infiltration showed an increase, the secretion of M1-related cytokines, including TNF-α and IL-6, also increased. The increase in these cytokines was accompanied by deterioration of renal function and elevation of kidney injury molecular-1 protein after 24 h of renal I/R. Some proteins, molecules, and transcription factors were involved in the M1 to M2 polarization, which helped reduce inflammation and trigger renal tubular cell repair [14,15,16]. The M2 macrophages were mainly involved in the late stages of I/R injury, which came with anti-inflammatory effects. Such effects mediated renal tubule repair. However, excessive M2 macrophages in the later stages of I/R injury could aggravate renal fibrosis by transitioning macrophage to myofibroblast. This results in the secretion of T1, ultimately disrupting the equilibrium between the tissue inhibitors of metalloproteinases (TIMPs) and matrix metalloproteinases (MMPs) while suppressing the degradation of the extracellular matrix (ECM) [17]. According to our findings (Figure 6 and Figure 7), CD206+ M2 macrophages infiltrate the kidney interstitium during late I/R injury. Alleviated infiltration of M2 macrophages by AS-IV results in reduced renal fibrosis during late I/R injury.

Recent studies indicated that AS-IV regulates cellular autophagy through multiple signaling pathways to exert an anti-inflammatory effect along with anti-hypoxic activity in different diseases [18,19,20]. AS-IV could also regulate the function of immune cells, especially macrophages, during the treatment of different disorders. In the process of skin damage caused by diabetes, AS-IV helps in reducing the levels of TNF-α, promoting the differentiation of macrophages to F4/80+CD206+ M2 macrophages, which accelerates skin wound healing [18]. AS-IV treatment reduces the mRNA levels of inflammatory factors (IL-1, IL-6, TNF-, COX-2, and iNOS) in LPS-mediated RAW 264.7 cells. [21] AS-IV provides a wide range of applications in kidney diseases. According to several studies, AS-IV protects against diabetic nephropathy by reducing podocyte apoptosis via the lncRNA-TUG1/TRAF5 signaling pathway. On the other hand, AS-IV also regulates the epithelial-mesenchymal transition (EMT) of podocytes via its regulatory effects on the Sirtuin 1 (SIRT1)/NF-κB pathway, activating autophagy [22,23]. AS-IV mainly affects the metabolic pathways related to inflammatory responses, oxidative stress, and energy metabolism [24,25,26]. Our experimental results showed that AS-IV exerted an anti-inflammatory and immunomodulatory effect. It aided renal function recovery, reduced macrophage infiltration, and suppressed the M1-associated proinflammatory cytokines, TNF-α and IL-6 in early stage of I/R It also elevated mRNA levels of IL-10 and Arginase I in M2-associated renal tissues. The preservation of renal function, reduction in kidney damage, and diminution of the inflammatory response were correlated to the phenotypic conversion of macrophages during acute kidney damage caused by IRI, which was attributed to the participation of AS-IV.

Ischemia-reperfusion is a pathological process that is represented as the coexistence of hypoxia and inflammation in the local renal tissues. The family of hypoxia-inducing factors (Hif-1α) plays a critical role in hypoxia by communicating with transcription factors and signaling molecules to increase their adaptability to the hypoxic microenvironment. Prolyl hydroxylase domain-containing protein (PHD1/2/3) blocks the hydroxylation of Hif-1α-α, which leads to its stability and binding to Hif-1α-β. It then translocates into the nucleus under acute hypoxia. The correlated signaling pathways, such as Akt, Nrf2, and mTOR, were reported to have crosstalk with Hif-1α [27,28,29]. The NF-κB represents the nuclear factor in classical inflammatory signaling pathways. The mutual reactivity between Hif-1α and NF-κB was discovered in 1994. During the inflammatory response, the consumption of oxygen by immune cells increases, further aggravating the environmental hypoxia and stabilizing Hif-1α. The promoters include hypoxia-responsive elements, which can activate NF-κB to the maximum extent. Hypoxia can up-regulate NF-κB independent of Hif-1α in both human and mouse macrophages [30]. Studies have shown that Hif-1α triggers NF-κB, which in turn controls the transcription of Hif-1α. NF-κB acts as a key transcriptional activator of Hif-1α in multiple cells under hypoxic conditions [31]. Hif-1α and NF-kB crosstalk is involved in many hypoxia-associated diseases, such as ischemic stroke, obstructive sleep apnea, and renal fibrosis [31,32,33]. We investigated the role of Hif-1α and NF-kB crosstalk in macrophage polarization and also explored whether AS-IV interfered with this mechanism. Our results showed the existence of a crosstalk between them, which affected macrophage polarization. The knockdown of NF-κB p65 downregulated the expression of Hif-1α. However, knockdown of Hif-1α did not interfere with the expression of p65 in oxygen-deprived macrophages. It is suggested that the expression of NF-κB p65 is involved in the regulation of the expression of Hif-1α. AS-IV stimulated the differentiation to M2 macrophages by suppressing the NF-κB/Hif-1α signaling pathway in hypoxic conditions. The modulation of AS-IV toward Hif-1α was found to be p65-dependent. Above all, this study revealed that AS-IV regulates M1 to M2 polarization, which reduces the proinflammatory response in order to protect the kidney from ischemia-reperfusion injury during the early stages (24 h after I/R).

Smad7 belongs to the SMAD family of proteins and can inhibit the TGF-β1 signaling pathway by preventing the formation of Smad2/Smad4 complexes [34]. It negatively regulates the Activin and bone morphogenetic protein (BMP) related pathways by occupying the type I receptors [35]. Smad7 was discovered in renal tubular epithelial cells (RTECs) during the inhibition of renal fibrosis [36]. Hif-1α has also been reported to have crosstalk with several pathways that promote fibrosis, such as Notch, PI3K/Akt, and Smad3 pathways during the process of renal fibrosis [37]. The TGF-β1/Smad3 pathway promotes the expression of Hif-1α within the RTECs [38], but the TGF-β1-mediated Hif-1α translation shows specificity for a few cell types. Within the mesangial cells, Hif-1α-2α was significantly induced by the TGF-β1/Smad3 signaling pathway instead of Hif-1α [39]. The role of Smad7 and Hif-1α in the macrophages was unknown in kidney I/R injury. Therefore, our study analyzed the effect of crosstalk between Hif-1α and Smad7 on M2 depolarization and also whether the treatment of AS-IV interfered with this mechanism. Our results showed that the knockdown of Hif-1α up-regulated the expression of Smad7, while the knockdown of Smad7 did not interfere with the expression of Hif-1α in oxygen-deprived macrophages. It is suggested that the expression of Hif-1α was involved in the regulation of Smad7. AS-IV inhibited secretion of TGF-β1 by suppressing the Hif-1α/Smad7 signaling pathway in hypoxic conditions. The modulation of AS-IV toward Smad7 was found to be dependent on Hif-1α. During the late stages of ischemia-reperfusion (28 days after I/R), the M2 macrophage infiltration leads to renal fibrosis. AS-IV alleviates the fibrosis through inhibition of M2 infiltration via the Hif-1α/Smad7 pathway.

## 5. Conclusions

In summary, AS-IV promoted the differentiation of M1 to M2, thereby relieving the proinflammatory response to alleviate the kidney injury during the early stages, while AS-IV attenuated M2 macrophage infiltration to prevent kidney fibrosis during the later stages.

## Figures and Tables

**Table 1:** Primer sequences for quantitative real-time polymerase chain reaction.

Gene		Sequences (5′-3′)	Length (bp)
TNF-α	Forward	accggcatggatctcaaagac	129 bp
	Reverse	gtccgggtgaggagcacgtagt	
IL-6	Forward	cccccaacttccaatgctctcct	134 bp
	Reverse	aggtcttgccgagtagacctc	
Arg1	Forward	aaagcctggtctgctggaaaa	122 bp
	Reverse	acagacccgtgggttcttcac	
IL-10	Forward	ccaagcccttatcggaaatga	111 bp
	Reverse	ttttcacagcgggagaaatcg	
β-actin	Forward	gccctgaggcctcttttccag	51 bp
	Reverse	tgccacaggacttccataccc	

## Data Availability

All data are available through email if reasonably requested.
